# Identification of potential hub genes associated with skin wound healing based on time course bioinformatic analyses

**DOI:** 10.1186/s12893-021-01298-w

**Published:** 2021-06-30

**Authors:** Hai-jun Zhu, Meng Fan, Wei Gao

**Affiliations:** The 4th People’s Hospital of Shenyang, No. 20 Huanghenan Street, Huanggu District, Shenyang, 110031 China

**Keywords:** Bioinformatic analysis, Split-thickness, Skin, Wound healing, Hub genes

## Abstract

**Background:**

The skin is the largest organ of the body and has multiple functions. Wounds remain a significant healthcare problem due to the large number of traumatic and pathophysiological conditions patients suffer.

**Methods:**

Gene expression profiles of 37 biopsies collected from patients undergoing split-thickness skin grafts at five different time points were downloaded from two datasets (GSE28914 and GSE50425) in the Gene Expression Omnibus (GEO) database. Principal component analysis (PCA) was applied to classify samples into different phases. Subsequently, differentially expressed genes (DEGs) analysis, Gene Ontology (GO) and Kyoto Encyclopedia of Genes and Genomes (KEGG) pathway functional enrichment analyses were performed, and protein–protein interaction (PPI) networks created for each phase. Furthermore, based on the results of the PPI, hub genes in each phase were identified by molecular complex detection combined with the ClueGO algorithm.

**Results:**

Using principal component analysis, the collected samples were divided into four phases, namely intact phase, acute wound phase, inflammatory and proliferation phase, and remodeling phase. Intact samples were used as control group. In the acute wound phase, a total of 1 upregulated and 100 downregulated DEGs were identified. Tyrosinase (TYR), tyrosinase Related Protein 1 (TYRP1) and dopachrome tautomerase (DCT) were considered as hub genes and enriched in tyrosine metabolism which dominate the process of melanogenesis. In the inflammatory and proliferation phase, a total of 85 upregulated and 164 downregulated DEGs were identified. CHEK1, CCNB1 and CDK1 were considered as hub genes and enriched in cell cycle and P53 signaling pathway. In the remodeling phase, a total of 121 upregulated and 49 downregulated DEGs were identified. COL4A1, COL4A2, and COL6A1 were considered as hub genes and enriched in protein digestion and absorption, and ECM-receptor interaction.

**Conclusion:**

This comprehensive bioinformatic re-analysis of GEO data provides new insights into the molecular pathogenesis of wound healing and the potential identification of therapeutic targets for the treatment of wounds.

**Supplementary Information:**

The online version contains supplementary material available at 10.1186/s12893-021-01298-w.

## Background

In humans, the skin is the largest, most exposed, and susceptible tissue. Wounds have become a significant healthcare problem due to the increasing number of trauma cases and pathophysiological conditions that clinicians treat [1]. To heal damaged skin, different cell types coordinate their action at precise stages. These complex, multi-step stages involve hemostasis, inflammation, re-epithelialization following keratinocyte migration and proliferation, and remodeling of the extracellular matrix (ECM), occurring in a temporally overlapping sequence [2]. Thus, skin repair is an elaborate process in humans. Numerous experimental studies have been conducted that have assisted in establishing a better understanding of the wound healing mechanism. However, the cellular and molecular mechanisms underpinning tissue repair and their failure to heal remain poorly understood, and thus current therapies are limited.

Partial-thickness skin grafts create a superficial wound at the donor site [3], characterized by a standardized depth of injury that extends to the epidermis and papillary dermis, which are renowned for their prolonged duration of healing, often resulting in a scar [4, 5]. Therefore, this type of wound is ideal for comparing superficial wound healing (WH) and scar formation in clinical experimental studies [6].

Microarray and high-throughput sequencing technologies provide genome-wide profiling of gene expression, allowing researchers to study WH in both animal models and humans. It also provides a large choice of gene sets with data representing the differential stages of normal WH [7]. In particular, time course studies allow researchers to study the dynamic behavior of gene expression, and consequently variations in molecular and cellular status over time [8]. A number of recent studies have used DNA microarrays to study the physiological and pathophysiological transcriptional response during WH [8, 9]. However, the biological information from these studies has not been fully explored. As bioinformatic technology rapidly advances, numerous data profiles from the GEO database have been reanalyzed by researchers.

In the present study, differentially expressed genes (DEGs) from two GEO datasets (GSE28914 [9] and GSE50425) in which intact and wounded skin samples over different phases of repair were compared, were reanalyzed. Principal component analysis (PCA) demonstrated that the expression of genes in each sample could be categorized into four separate phases. We hypothesized that those four phases related to the four emblematic periods of WH, including intact tissue, acute wound phase, inflammation, and the remodeling phase. Furthermore, using bioinformatic methods, the integrated DEGs within each phase were identified, followed by Gene Ontology (GO) and Kyoto Encyclopedia of Genes and Genomes (KEGG) pathway enrichment analyses. Subsequently, an analysis of PPI and hub genes was also performed.

## Methods

### Microarray data information

The gene expression profiles of patients undergoing split-thickness skin graft harvesting biopsies (Accession nos. GSE28914 and GSE50425) were obtained from the Gene Expression Omnibus (GEO) database (http://www.ncbi.nlm.nih.gov/geo/). The data were downloaded and annotated using “GEOquery” [10] and “Bioconductor” [11] R packages using the R language platform (version 3.5.1). The genomics platforms used were the GPL570 [HG-U133_Plus_2] Affymetrix Human Genome U133 Plus 2.0 Array for GSE28914 and GPL10558 Illumina HumanHT-12 V4.0 expression BeadChip for GSE50425.

The GSE28914 dataset represents 25 biopsies, of which 8 are from samples of intact skin (IS), 6 from acute wounds (AW), 6 from the third-postoperative-day (3rd POD), and 5 from the seventh-postoperative-day (7th POD) from 8 different male patients undergoing split-thickness skin graft harvesting. The GSE50425 dataset represents the data from 12 biopsies, including 4 from each of samples of intact skin (IS), the fourteenth-postoperative-day (14th POD), and the twenty-first-postoperative-day (21st POD) from four split-thickness skin graft donors. The IS samples in each array were considered as controls. All data are freely accessible and were created without the involvement of any additional human or animal experiments.

### PCA of gene expression

In the present study, the quality of each microarray data was evaluated by conducting PCA [12] prior to analysis of the DEGs. The ‘pca3d’ algorithm in R language was used to evaluate the PCA of gene expression, resulting in a 3-dimensional graph demonstrating which DEGs were considered as variables and showing the differences between IS and wound samples at different time points.

### Identification of DEGs

The DEGs between POD and IS samples were identified by adjusting the selection criteria of the DEGs for just those with P-values (adj-P) ≤ 0.05 and |log2FC|≥ 2. Both the normalization of data and the screening of DEGs were performed using the linear models of microarray data (limma) package (http://bioconductor.org/packages/release/bioc/html/limma.html) within the R language environment. The DEGs scripts of each phase used in the analysis was presented in Additional file 2

### GO and KEGG pathway enrichment analysis of DEGs

The role of DEGs in WH was established following the analysis of all DEGs using the DAVID network portal (https://david.ncifcrf.gov/) [13] and their biological attributes extracted, including data about which biological processes (BP), molecular functions (MF), and cellular components (CC) were involved.

ClueGO, a widely used Cytoscape plugin allowing visualization of nonredundant biological terms for large clusters of genes in a functionally grouped network [14], was used to decipher functionally grouped KEGG pathway annotations, using the ‘fusion’ feature for data where P < 0.05. Finally, bar graphs and bubble plots of the results of functional enrichment were created using the “ggplot2” R package (version 3.2.0; CRAN.R-project.org/package=ggplot2) in the R language environment. The scripts of KEGG pathway enrichment analysis of each phase used in the analysis was presented in Additional file 3, and the scripts of GO enrichment analysis of each phase used in the analysis was presented in Additional file 4.

### PPI network and hub gene analysis

A PPI network of all DEGs was plotted using the Search Tool for the Retrieval of Interacting Genes (STRING) (http://string-db.org/) tool to evaluate all interactional associations of the proteins. The PPI network was visualized using Cytoscape software (v3.7.2). To identify high-level genes that play a key role in PPI network, the Cytohubba package based on Cytoscape was used to perform the hub gene analysis [24], and the top rank 10 genes in all modules are considered to be the hub genes. The degree of all nodes in PPI network was calculated using the Cytoscape plugin MCODE and the most significant cluster was selected. ‘Maximum number of interactors = 0’ and ‘confidence score ≥ 0.4’ were selected as cut-off criteria. To further clarify the identity of the genes in most significant cluster, KEGG analysis was performed using ClueGO with the kappa score adjusted to reflect the relationship between terms where there was a similarity in related genes [15]. Each gene that represented a link in multiple pathways was identified as a key functional gene. The scripts of PPI network plotted by STRING of each phase used in the analysis were presented in Additional file 5.

## Results

### Validation of the datasets

**Fig. 1:**
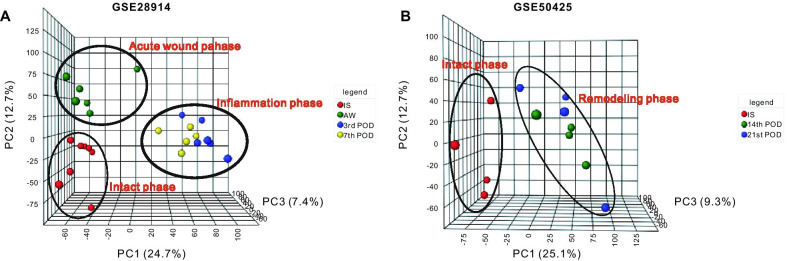
Principal component analysis (PCA) of differentially expressed genes (DEGs) between intact skin samples and the wound sample at different phases. Genes were plotted in 3D visualization, indicating that samples within each group shared more similarity. **A** The red dots showed the PCA values of 8 intact skin samples (IS), and the green dots indicate the PCA value of 6 acute wounds samples (AW), and the blue dots indicate the PCA value of 6 third-postoperative-day samples (3rd POD), and the yellow dots indicate the PCA value of 5 seventh-postoperative-day samples (7th POD) from GSE28914. **B** The red dots showed the PCA values of 4 intact skin samples (IS), and the green dots indicate the PCA value of 4 fourteenth-postoperative-day samples (14th POD), and the blue dots indicate the PCA value of 4 twenty first-postoperative-day samples (21st POD) from GSE50425

The robust multi-array average (RMA) algorithm was used to display the distribution of test values after quantile normalization, background correction, and quartile data normalization of the downloaded data [16]. The results demonstrated that following normalization, the median of the different samples was almost the same value, indicating a high degree of standardization (Additional file 1A and B). To further validate intra-group data repeatability, PCA was employed to evaluate the data distribution in each sample to guarantee that the data was accurate and reliable. The evaluation suggested that the data from IS and all samples periods up to 21 days post skin injury could be partitioned into four distinct transcriptional phases, namely intact phase, acute wound phase, inflammation, and remodeling phase (Fig. 1A, B).

### Bioinformatic analysis of the acute wound phase

**Fig. 2:**
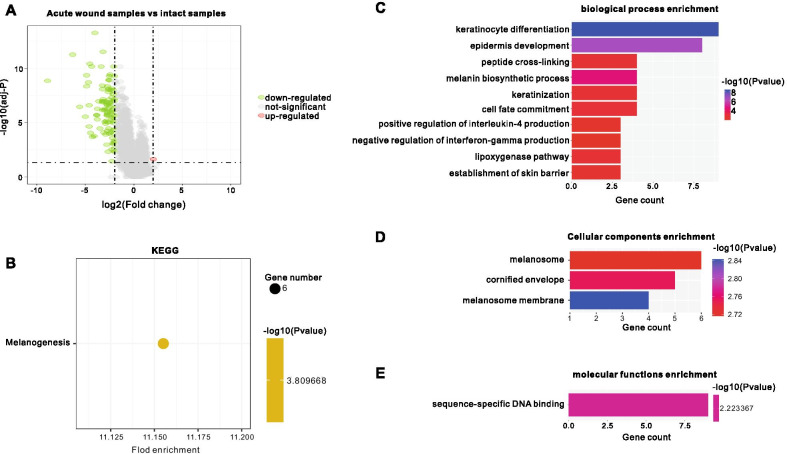
Differentially expressed genes (DEGs) in the acute wound phase, and Gene Ontology (GO) and KEGG pathway analysis of DEGs. **A** Volcano plot representing DEGs in acute wound samples compared with intact skin samples (control). **B** The most significantly enriched KEGG pathways and numbers of genes involved (*P* < 0.01); **C** The most significantly enriched (*P* < 0.01) GO terms and numbers of genes involved in acute wound samples for biological processes, **D** cellular components, **E** molecular functions, compared to the control tissue

Volcano plot analysis was used to present the DEGs in AW samples compared with intact samples (Fig. 2A). A total of 1 upregulated and 100 downregulated genes were identified in AW samples compared with IS.

DEG functional annotation was conducted using the DAVID network portal. A threshold of *P* < 0.001 selected for KEGG analysis identified enrichment in only one critical pathway, melanogenesis (Fig. 2B). Bar graphs of DEGs GO enrichment demonstrate the distribution of enriched GO terms in BP, CC, and MF categories. The most significantly enriched terms for each category is presented in Fig. 2C–E, for which a threshold of *P* < 0.001 was selected. The results demonstrate that variations in DEGs linked with BP were mostly those enriched in keratinocyte differentiation, epidermis development, and melanin biosynthetic process (Fig. 2C). Variations in DEGs linked with CC were significantly enriched in melanosome membrane, cornified envelope, and melanosome (Fig. 2D). For MF, DEGs were only significantly enriched in sequence-specific DNA binding (Fig. 2E).

**Fig. 3:**
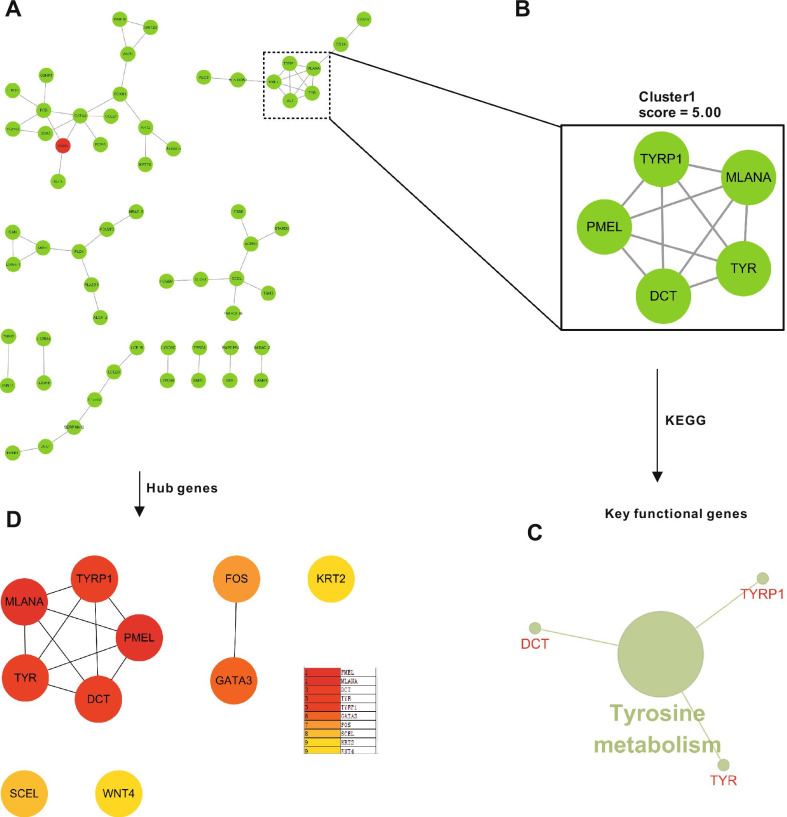
Hub clusters of DEGs in the acute wound phase analyzed by protein–protein interaction (PPI) analysis and hub genes related to KEGG pathway enrichment. **A** PPI interaction network. Green nodes represent downregulated genes; red nodes represent upregulated genes. **B** PPI molecular complex detection method (MCODE) component. The highest scoring module identified from the PPI network using the MCODE algorithm with a score of 5.00, with five key genes involved. **C** Key genes in cluster 1 were annotated in the context of the KEGG database by ClueGO, of which 3 (TYR, TYRP1, and DCT) were enriched in the tyrosine metabolism pathway, and defined as key functional genes. **D** The top 10 hub genes in the PPI were screened by Cytoscape plugin cytoHubba based on their connectivity degree

To explore the biological characteristics of these DEGs, a PPI network was created using the STRING database. The PPI network consisted of 59 edges and 98 nodes after pairs were isolated from the major network (Fig. 3A). The PPI network was additionally analyzed through the use of the MCODE algorithm which indicated that the highest score cluster contained five key genes, including Melan-A (MLANA), tyrosinase (TYR), tyrosinase Related Protein 1 (TYRP1), dopachrome tautomerase (DCT), and premelanosome protein (PMEL) (Fig. 3B). Additional KEGG enrichment analysis of those key genes resulted in the identification of three key functional genes (TYR, TYRP1, and DCT) involved in tyrosine metabolism which dominate the process of melanogenesis (Fig. 3C). Subsequently, the Hub genes from the PPI network were analyzed by using the MCC algorithm in the CytoHuba plugin, and top 10 Hub genes (Fig. 3D) were ranked based on the MCC scores, which were PMEL, MLANA, DCT, TYR, TYRP1, GATA Binding Protein 3 (GATA3), Fos Proto-Oncogene (FOS), Sciellin (SCEL), Keratin 2 (KRT2) and Wnt Family Member 4 (WNT4).

### Bioinformatic analysis of the inflammatory and proliferation phase

**Fig. 4:**
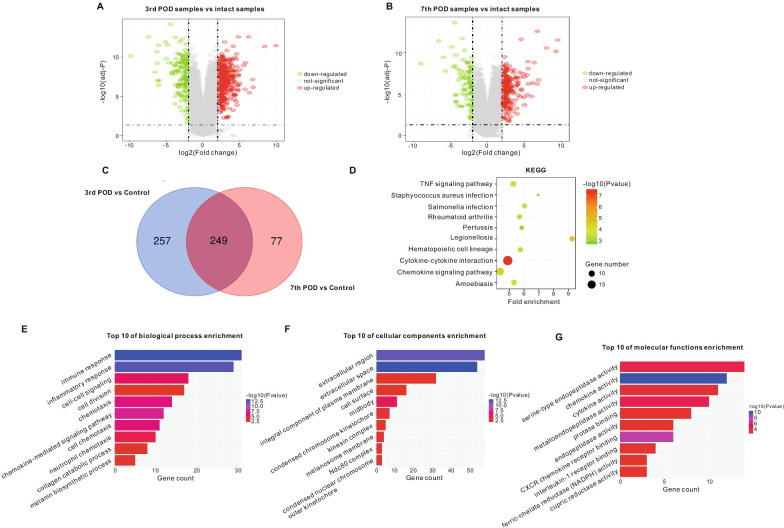
Differentially expressed genes (DEGs) in the inflammatory phase, and Gene Ontology (GO) and KEGG pathway analysis of DEGs. **A** Volcano plot representing DEGs on the third-postoperative-day (3rd POD) compared with intact skin (control). **B** Volcano plot representing DEGs on the seventh-postoperative-day (7th POD) compared with control. **C** Venn diagram displaying overlapping genes that were significantly differentially expressed on the 3rd and 7th POD, compared with the control. **D** Ten most significantly enriched KEGG pathways and numbers of genes involved; **E** Ten most significantly enriched GO terms for biological processes and numbers of genes involved; **F** Ten most significantly enriched GO terms for cellular components and numbers of genes involved gene numbers; **G** Ten most significantly enriched GO terms for molecular function of DEGs and numbers of genes involved, in the significantly differentially expressed overlapping genes on the 3rd and 7th POD compared with the control. P < 0.05 defined as significantly enriched

Volcano plot analysis was conducted to display the DEGs within the 3rd and 7th POD samples compared with IS in GSE28914 (Fig. 4A, B). In total, 360 upregulated and 146 downregulated genes were identified in 3rd POD samples, and 232 upregulated and 94 downregulated genes in 7th POD samples, compared with IS. Genes in the 3rd and 7th POD samples were chummy in PCA. A total of 249 overlapping DEGs, including 85 upregulated and 164 downregulated genes were identified in the two databases (Fig. 4C).

Using the DAVID portal, functional annotation was performed on overlapping DEGs. A threshold of *P* < 0.01 was selected from which the most significant KEGG pathways were determined. These DEGs were enriched in cytokine–cytokine receptor interaction, legionellosis, chemokine signaling pathway, amoebiasis, TNF signaling pathway, salmonella infection, hematopoietic cell lineage, rheumatoid arthritis, pertussis, and staphylococcus aureus infection (Fig. 4D). The enriched functions of the overlapping DEGs identified in GO analysis for BP were mainly those of immune response, inflammatory response, chemokine-mediated signaling pathway, chemotaxis, cell chemotaxis, cell–cell signaling, neutrophil chemotaxis, melanin biosynthetic process, cell division, and collagen catabolic process (Fig. 4E). The overlapping DEGs linked with CC were significantly enriched in extracellular space, extracellular region, midbody, melanosome membrane, condensed chromosome kinetochore, Ndc80 complex, condensed nuclear chromosome outer kinetochore, an integral component of the plasma membrane, kinesin complex, and cell surface (Fig. 4F). In terms of MF, the overlapping DEGs were significantly enriched in chemokine activity, CXCR chemokine receptor binding, metalloendopeptidase activity, serine-type endopeptidase activity, cytokine activity, protease binding, interleukin-1 receptor binding, endopeptidase activity, ferric-chelate reductase (NADPH) activity, and cupric reductase activity (Fig. 4G).

**Fig. 5:**
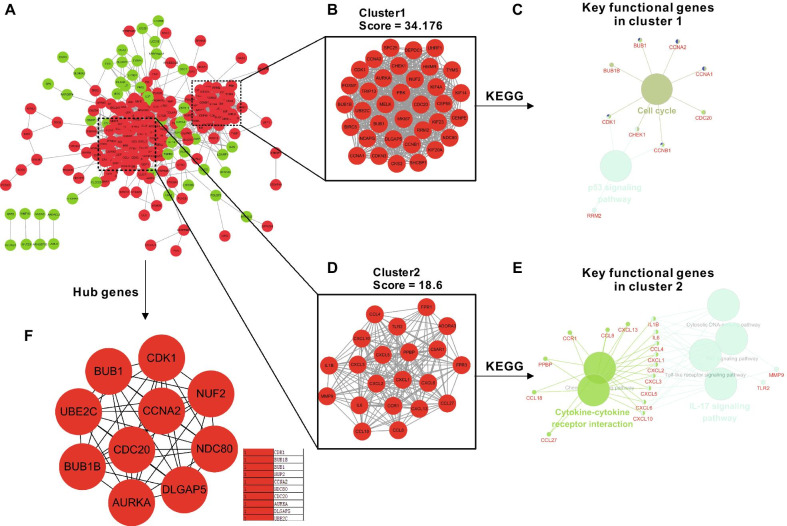
Hub cluster of DEGs in the inflammatory phase analyzed by protein–protein interaction (PPI) and hub genes related to KEGG pathway enrichment. **A** PPI interaction network. Green nodes represent downregulated genes; red nodes represent upregulated genes. PPI molecular complex detection method (MCODE) component. Two highest-scoring modules identified in the PPI network using the MCODE algorithm with scores of 34.176 (cluster 1) and 18.6 (cluster 2). **B** In cluster 1, 35 key genes were involved, and **D** 21 key genes in cluster 2 were involved. Key genes in cluster 1 (**C**) and cluster 2 (**E**) were annotated in the context of the KEGG database. The relationships between these annotated terms were calculated and grouped by ClueGO to create an annotation module network. Genes with connections having more than two terms were defined as key functional genes. **F** The top 10 hub genes in the PPI were screened by Cytoscape plugin cytoHubba based on their connectivity degree

After the pairs were isolated from the entire network, the PPI network of shared DEGs comprised 1504 edges and 246 nodes (Fig. 5A). The PPI network of DEGs was further analyzed using the MCODE algorithm, in which two high-scoring clusters were found. In cluster 1, the score as judged by the algorithm was 34.176, which included 35 key genes (Fig. 5B). In cluster 2, the score judged by the algorithm was 18.6, for which 21 key genes were included (Fig. 5D). The key genes in each cluster identified by KEGG pathway enrichment were re-analyzed using the “ClueGO” and “CluePedia” plugins for Cytoscape software (P < 0.05). The results indicated that the key functional genes in cluster 1 were checkpoint kinase 1 (CHEK1), CyclinB1 (CCNB1) and cyclin-dependent kinases 1 (CDK1), which were substantially enriched in cell cycle and P53 signaling pathway (Fig. 5C). The key functional genes in cluster 2 were interleukin (IL) 1B, IL6, C–C Motif Chemokine Ligand (CCL) 4, C-X-C motif chemokine (CXCL) 1, CXCL2, CXCL3, CXCL5, CXCL6, and CXCL10, which were considerably enriched in both cytokine–cytokine receptor interaction and IL-17 signaling pathway (Fig. 5E). Subsequently, the Hub genes from the PPI network were analyzed by using the MCC algorithm in the CytoHuba plugin, and top 10 Hub genes were ranked based on the MCC scores (Fig. 5F), which were CDK1, BUB1 Mitotic Checkpoint Serine/Threonine Kinase B (BUB1B), BUB1 Mitotic Checkpoint Serine/Threonine Kinase (BUB1), NUF2 Component Of NDC80 Kinetochore Complex (NUF2), Cyclin A2 (CCNA2), NDC80 Kinetochore Complex Component (NDC80), Cell Division Cycle 20 (CDC20), Aurora Kinase A (AURKA), DLG Associated Protein 5 (DLGAP5) and Ubiquitin Conjugating Enzyme E2 C (UBE2C).

### Bioinformatic analysis of the remodeling phase

**Fig. 6:**
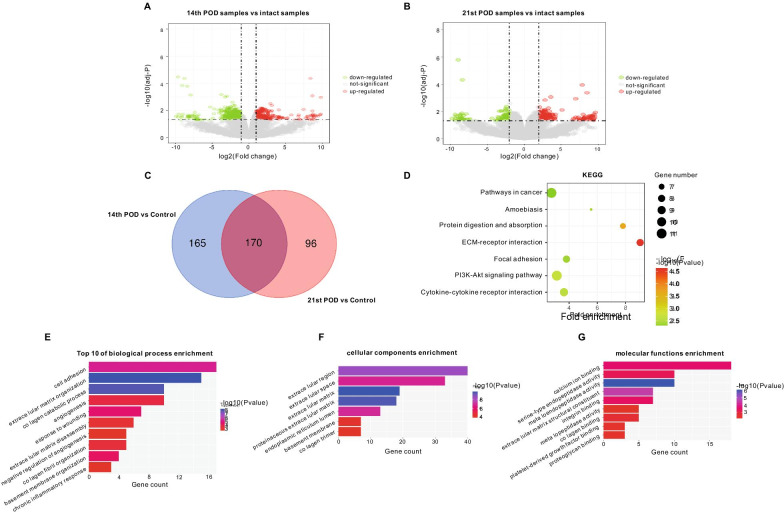
Differentially expressed genes (DEGs) in the remodeling phase, and Gene Ontology (GO) and KEGG pathway analysis of DEGs. **A** Volcano plot representing DEGs on the fourteenth-postoperative-day (14th POD) compared with intact skin (control). **B** Volcano plot representing DEGs on the twenty first-postoperative-day (21st POD) compared with the control. **C** Venn diagram displaying significantly differentially expressed overlapping genes on the 14th and 21st POD compared with the control. **D** The most significantly enriched KEGG pathways and numbers of genes involved; **E** Ten most significantly enriched GO terms for biological processes and numbers of genes involved; **F** The most significantly enriched GO terms for cellular components and numbers of genes involved; **G** The most significantly enriched GO terms for molecular function of DEGs and numbers of genes involved in the overlapping genes that were significantly differentially expressed on the 14th and 21st POD compared with control. *P* < 0.01 defined as significantly enriched

Volcano plot analysis was conducted to display the DEGs in the 14th and 21st POD samples compared with IS from GSE50425, respectively. In total, 213 upregulated and 122 downregulated genes were identified in the 14th POD samples and 170 upregulated and 96 downregulated genes in the 21st POD samples compared with the IS (Fig. 6A, B). Since gene expression of the 14th and 21st POD samples were clustered when analyzed by PCA, a total of 170 overlapping DEGs, including 121 upregulated and 49 downregulated genes were identified in the two databases (Fig. 6C).

Using the DAVID network portal, functional annotation was performed on the overlapping DEGs. Using a threshold of *P* < 0.01, KEGG pathways were significantly enriched in pathways in cancer, amoebiasis, protein digestion and absorption, ECM-receptor interaction, focal adhesion, PI3K–Akt signaling pathway and cytokine–cytokine receptor interaction (Fig. 6D). From GO analysis of top 10 BPs, the overlapping DEGs were mainly functionally enriched in cell adhesion, extracellular matrix organization, collagen catabolic process, response to wounding, basement membrane organization, angiogenesis, collagen fibril organization, extracellular matrix disassembly, and negative regulation of angiogenesis and the chronic inflammatory response (Fig. 6E). Differences in the overlapping DEGs linked with CC were significantly enriched in the extracellular region, extracellular space, extracellular matrix, proteinaceous extracellular matrix, endoplasmic reticulum lumen, basement membrane and collagen trimer (Fig. 6F), while for MF, the overlapping DEGs were significantly enriched in calcium ion binding, serine-type endopeptidase activity, extracellular matrix structural constituent, integrin binding, metalloendopeptidase activity, collagen binding, platelet-derived growth factor binding and proteoglycan binding (Fig. 6G).

**Fig. 7:**
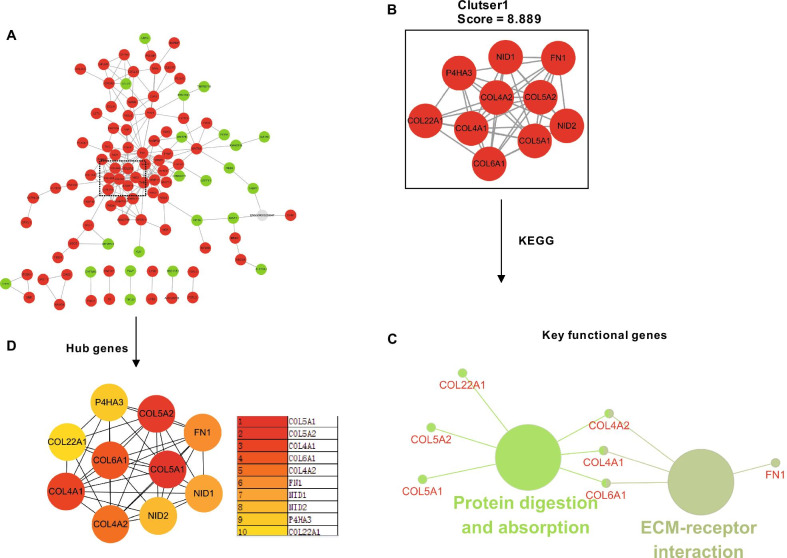
Hub clusters of DEGs in the remodeling phase analyzed by protein–protein interactions (PPI) and hub genes related to KEGG pathway enrichment. **A** PPI interaction network. Green nodes represent downregulated genes; red nodes represent upregulated genes. **B** PPI molecular complex detection method (MCODE) component. Highest scoring module identified from the PPI network using the MCODE algorithm with a score of 8.889 (cluster 1), with five key genes involved. **C** Key genes in the cluster were annotated in the context of the KEGG database and the relationships between these annotated terms were calculated and grouped using ClueGO to create an annotation module network. Genes with connections to more than two terms were defined as hub genes. **D** The top 10 hub genes in the PPI were screened by Cytoscape plugin cytoHubba based on their connectivity degree

After the pairs were isolated from the complete network, the PPI network of overlapping DEGs comprised 201 edges and 168 nodes (Fig. 7A). The PPI network of DEGs was further analyzed using the MCODE algorithm, with the highest score cluster containing ten key genes (Fig. 7B). To elucidate the potential pathway of the key genes within the cluster, KEGG pathway enrichment was further analyzed using the “ClueGO” and “CluePedia” plugins in Cytoscape software (*p* < 0.05). The results indicated that seven genes were involved in protein digestion and absorption, and ECM-receptor interaction. Three key functional genes, COL4A1, COL4A2, and COL6A1, connected those two pathways (Fig. 7C). Subsequently, the Hub genes from the PPI network were analyzed by using the MCC algorithm in the CytoHuba plugin, and top 10 Hub genes were ranked based on the MCC scores (Fig. 7D), which were COL5A1, COL5A2, COL4A1, COL6A1, COL4A2, Fibronectin 1 (FN1), Nidogen 1 (NID1), Nidogen 2 (NID2), Prolyl 4-Hydroxylase Subunit Alpha 3 (P4HA3) and COL22A1.

## Discussion

The normal WH process includes a well-orchestrated and regulated process consisting of a series of events, namely hemostasis, inflammation, proliferation, and ECM remodeling [2]. Various studies have been conducted on WH, although the specific molecular mechanisms occurring within each phase remain elusive. Therefore, it is crucial that the pivotal molecules playing critical roles in the pathogenesis of WH are identified so that potential therapeutic targets can be developed, and thus this represents an important area of investigation. Gene expression microarrays provide a comprehensive analysis of genome-wide expression profiles of clinical samples and have been widely used to explore potential therapeutic targets in a variety of diseases [17, 18].

In 2012, Kristo Nuutila and colleagues used genome-wide expression profiling to investigate time-course gene expression in epidermal wounds via the use of 25 split-thickness skin graft biopsies from 8 healthy adult patients (GSE28914) [9]. The samples were collected from graft donor site wounds promptly before and after harvesting, in addition to during the healing process, on the third and seventh day. Furthermore, in 2013, the same team collected 14 biopsies from patients undergoing split-thickness skin graft harvesting (GSE50425), from intact skin and from their wounds on the 14th and 21st post-operative days. However, in these studies, only the fold change in DEGs was reported and no functional analysis data was evaluated. With the recent development of bioinformatic technology, the comprehensive analysis of microarray data from multiple centers has become a focus of research attention. Thus, in the present study, the gene expression profiles from the laboratory of Kristo Nuutila (GSE28914 and GSE50425) were downloaded and reanalyzed. Using bioinformatic methods, integrated DEGs were identified, and GO and KEGG pathway enrichment analysis performed. Analysis of PPI networks and hub genes was also subsequently conducted.

Previous studies have reported that the process of WH can be categorized into three to five distinct phases that occur sequentially over time [19, 20]. In the present study, reanalysis of the microarray expression data demonstrated, through the use of PCA, that the WH process could be divided into four phases. We hypothesized that the four phases were intact phase, acute wound phase, inflammatory and proliferation phase, and remodeling phase (Fig. 1). DEGs were reanalyzed in each phase from split-thickness skin graft biopsies at different healing time points.

The first phase of physiological or acute WH is hemostasis and the formation of a provisional wound matrix occurring promptly after injury and completed within a few hours [20]. Our KEGG enrichment results of acute wound phase, principally concerning downregulated genes, included those involved in melanogenesis pathway when *P* < 0.01 (Fig. 2B). Although the P-values of the signaling pathways regulating pluripotency of stem cells and the Hippo signaling pathway were 0.03 and 0.04, not very much different (Additional file 3). Stem cells play an essential role in WH and have been widely studied, they also participate in accelerating the recovery of skin. Additionally, no hemostasis-related pathway was identified in the KEGG enrichment analysis, possibly supporting the notion that physiological capillary rupture following skin injury may not be required [21, 22]. In addition, the GO analysis in present study demonstrated that the biological process of keratinocyte differentiation was initiated early in the acute wound phase (Fig. 2C), consistent with previous reports that keratinocyte migration is an early event in wound re-epithelialization [23, 24].

Subsequent analysis clarified the proposition that tyrosine metabolism is the key pathway during the initial stage of WH, with three key functional genes, TYR, TYRP1 and DCT, that are involved (Fig. 3C). TYR, which converts tyrosine to dopaquinone, is the key enzyme involved in the rate-limiting step of tyrosine metabolism, and TYRP1 is an important melanosomal enzyme belonging to the TYR family. DCT is an important paralog of TYRP1 [25]. Similarly, TYR, TYRP1, and DCT in this study were all significantly down-regulated in the acute wound samples compared with intact skin. Interestingly, combined with Hub genes analysis, we found that DCT, TYR, TYRP1 are both Hub and keg functional genes. This possibly suggests that the inhibition of tyrosine metabolism may play an important role in the initial stage of skin repair. Combined with the KEGG results of acute wound phase, we speculated that targeting TYRP1 or DCT to inhibit the tyrosine metabolic pathway in keratinocyte will become a potential therapeutic strategy for alleviating or treating acute wound. Moreover, this speculate has been implemented in the recent studies [26, 27], but there is no clinical research.

The inflammatory phase begins with edema due to increased vascular hyperpermeability, generally occurring 72 h following skin injury [28]. We analyzed overlapping DEGs in the samples harvested on the third and seventh day after skin injury and compared them to the control group. Interestingly, from a total of 249 DEGs, the trend in expression of all overlapping DEGs remained the same on the 3rd and 7th POD (data not shown). Subsequently, the GO terms and KEGG pathways of the overlapping DEGs that were significantly enriched were found to be closely associated with the immune response, especially the chemokine signaling pathway, as found in previous studies [2, 29].

Interestingly, when the high-scoring clusters in the PPI network were analyzed using MCODE combined with the ClueGO algorithm, we found that the highest scoring cluster was not immune-related, but cell cycle-related (Fig. 5B–E). Cell proliferation is an essential feature of immune cell activation. The p53 signaling pathway is a classic cell cycle regulatory pathway applicable in multiple cell types. The results of the present study demonstrated that CHEK, CCNB1, and CDK1 play vital roles in this phase. Furthermore, combined with Hub genes analysis, only CDK1 was found to be the Hub and key functional gene.

In addition, considerable effort has been expended over recent decades to identify the impact of each cytokine on various parameters of WH in diverse experimental models. The results here reveal that the key functional genes include IL1B, IL-6, CCL4, CXCL1, CXCL2, CXCL3, CXCL5, CXCL6, and CXCL10, which can co-express and play essential roles in cytokine–cytokine receptor interactions and the IL-17 signaling pathway. Of these, the majority are chemokine receptors, except for IL-1B and IL-6, which are pro-inflammatory cytokines. A previous study found that IL-6 knockout mice suffered delayed healing. The results of the present study reveal that upregulated IL-6 expression is key in skin healing, consistent with phenotypes observed in IL-6 knockout mice [30]. Another study also found that IL-1B and IL-6 were elevated significantly during healing and could increase keratinocyte motility in wounds [31]. Furthermore, a different study also found that low expression of CXCL1 and CXCL5, both of which are chemoattractant for neutrophils, inhibited mouse WH [32].

The third phase of wound healing is remodeling which begins 2 to 3 weeks after the onset of the lesion and can last for a year or more [2]. The core purpose of this stage is to achieve maximum tensile strength through reorganization, degradation, and resynthesis of the extracellular matrix [2]. In the final stage of healing, some attempt to restore normal tissue structure occurs, with the gradual remodeling of the granulation tissue, resulting in scar tissue that is less cellular and vascular, exhibiting a progressive increase in the concentration of collagen fibers [33]. In the present study, the essential terms from the KEGG and GO enrichment analysis in this phase were ECM-receptor interaction pathway and cell adhesion biological process (Fig. 6), consistent with previous research. The BP enrichment further showed that collagen, the most abundant component of the basement membrane organization, plays the critical role. Beside, we also found that cytokine–cytokine receptor interaction continues to play a role, even on the 21st POD, suggesting that inflammatory still play an important role in this phase.

To further explore potential targets, we identified COL4A1, COL4A2, and COL6A1 as key functional genes which co-express both with protein digestion and absorption and ECM-receptor interaction (Fig. 6C). Together with Hub gene analysis, results showed that COL4A1, COL4A2, and COL6A1 are both Hub and key functional genes. The proteins encoded by COL4A1 and COL4A2 are two of the six subunits of type IV collagen. The protein encoded by COL6A1 is the alpha 1 subunit of type VI collagen. Although 28 types of collagen have been identified, collagens I and III, comprise approximately 90% and 10% of the total collagen in the skin. However, less prevalent collagen types are also essential for normal skin function. Type IV collagen is a type in skin found primarily within the basement membrane zone. One study demonstrated that type IV collagen could be observed through the use of immunolocalization around the cutaneous sensory nerve, blood vessels, and sweat glands [34, 35]. Unfortunately, the mechanism by which collagen IV achieves wound-healing in the skin remains elusive [33]. Type VI collagen is a non-fibrillar form expressed in many connective tissues and involved in the organization of matrix, and contributing to tissue remodeling [36]. Mutations in any of the three genes that encode the type VI collagen chains (COL6A1, COL6A2, and COL6A3) can cause disorders that affect muscle and connective tissue, with such clinical features as muscular weakness, joint contractures, and laxity [37]. However, another study with Col6a1 null mice found that Col6a1 deficiency did not result in a visible WH defect, although it resulted in decreased tensile strength of the skin and an altered collagen fibril and basement membrane architecture [38]. Thus, we speculated that the overexpression of COL4A1 and COL4A2 and resulting in type IV collagen hyperplasia is the cause of scar tissue formation.

In summary, this comprehensive GEO bioinformatic data re-analysis provides new insights into the molecular pathogenesis of WH and the identification of potential therapeutic targets for the treatment of each phase of wounds. The present study highlights co-expression gene networks associated with WH in each phase. However, further study, such as the analysis of single-cell sequencing data is required to establish the precise identity of hub genes in the specific cell types in WH. Moreover, the main limitation of this study is the lack of experimental verification. In the future, it will be of great significance to conduct a further systematic study on those hub and key functional genes to investigate the detailed mechanisms.

## Conclusions

The present study systematically investigated multiple microarray gene expression profiles. The identity of hub genes at different time points was closely associated with the onset, development, and prognosis of WH. However, future studies are required to elucidate the biological function of these genes in WH.

## Supplementary Information

**Additional file 1.** Post-standardization values of gene expression in (A) GSE28914 and (B) GSE50425 are presented as boxplots.

**Additional file 2.** DEGs in each phase.

**Additional file 3.** KEGG pathway enrichment analysis of DEGs in each phase.

**Additional file 4.** GO enrichment analysis of DEGs in each phase.

**Additional file 5.** STRING data for PPI network in each phase.

## Data Availability

The raw data of GSE28914 and GSE50425 are available from the Gene Expression Omnibus (GEO) database (http://www.ncbi.nlm.nih.gov/geo/). The datasets analysed during the current study are almost in the additional tables. If there are any other questions, please contact the corresponding author by email.

## Abbreviations

GEO: Gene Expression Omnibus
PCA: Principal component analysis
DEGs: Differentially expressed genes
GO: Gene Ontology
KEGG: Kyoto Encyclopedia of Genes and Genomes
PPI: Protein–protein interaction
MCC: Maximal Clique Centrality
ECM: Extracellular matrix
AW: Acute wounds
POD: Postoperative-day
IS: Intact skin
BP: Biological processes
MF: Molecular functions
CC: Cellular components
STRING: Search Tool for the Retrieval of Interacting Genes
RMA: Robust multi-array average
TYR: Tyrosinase
TYRP1: Tyrosinase Related Protein 1
DCT: Dopachrome tautomerase
MLANA: Melan-A
PMEL: Premelanosome protein
CHEK1: Checkpoint kinase 1
CDK1: Cyclin-dependent kinases 1
IL: Interleukin
CXC: C-X-C motif chemokine
FOS: Fos Proto-Oncogene
SCEL: Sciellin
KRT2: Keratin 2
WNT4: Wnt Family Member 4
BUB1B: BUB1 Mitotic Checkpoint Serine/Threonine Kinase B
BUB1: BUB1 Mitotic Checkpoint Serine/Threonine Kinase
NUF2: NUF2 Component of NDC80 Kinetochore Complex
CCNA2: Cyclin A2
NDC80: NDC80 Kinetochore Complex Component
CDC20: Cell Division Cycle 20
AURKA: Aurora Kinase A
DLGAP5: DLG Associated Protein 5
UBE2C: Ubiquitin Conjugating Enzyme E2 C
FN1: Fibronectin 1
NID1: Nidogen 1
NID2: Nidogen 2
P4HA3: Prolyl 4-Hydroxylase Subunit Alpha 3
